# Is cancer an intelligent species?

**DOI:** 10.1007/s10555-023-10123-0

**Published:** 2023-08-04

**Authors:** Chiara Nicolazzo, Federica Francescangeli, Valentina Magri, Alessandro Giuliani, Ann Zeuner, Paola Gazzaniga

**Affiliations:** 1 Department of Molecular Medicine, Sapienza University of Rome, 00161 Rome, Italy; 2 Department of Oncology and Molecular Medicine, Istituto Superiore di Sanità, Viale Regina Elena 299, 00161 Rome, Italy; 3 Department of Pathology, Oncology and Radiology, Sapienza University of Rome, 00161 Rome, Italy; 4 Environment and Health Department, Istituto Superiore di Sanità, Viale Regina Elena 299, 00161 Rome, Italy

**Keywords:** Intelligence, Adaptation, Circulating tumor cells, EMT

## Abstract

Some relevant emerging properties of intelligent systems are “adaptation to a changing environment,” “reaction to unexpected situations,” “capacity of problem solving,” and “ability to communicate.” Single cells have remarkable abilities to adapt, make adequate context-dependent decision, take constructive actions, and communicate, thus theoretically meeting all the above-mentioned requirements. From a biological point of view, cancer can be viewed as an invasive species, composed of cells that move from primary to distant sites, being continuously exposed to changes in the environmental conditions. Blood represents the first hostile habitat that a cancer cell encounters once detached from the primary site, so that cancer cells must rapidly carry out multiple adaptation strategies to survive. The aim of this review was to deepen the adaptation mechanisms of cancer cells in the blood microenvironment, particularly referring to four adaptation strategies typical of animal species (phenotypic adaptation, metabolic adaptation, niche adaptation, and collective adaptation), which together define the broad concept of biological intelligence. We provided evidence that the required adaptations (either structural, metabolic, and related to metastatic niche formation) and “social” behavior are useful principles allowing putting into a coherent frame many features of circulating cancer cells. This interpretative frame is described by the comparison with analog behavioral traits typical of various animal models.

## About species and intelligence: a necessary premise

Although the question of what constitutes a species has a long, argumentative history, a universal definition of species is far from being established. Settling whether cancer is a species in its own is beyond the scope of this review; nevertheless, the title we have chosen forces us to make a little reflection. Since various concepts of species, some mutually exclusive, some other interchangeable, have followed one another over time, it is particularly hard to answer whether cancer can be considered as a species. Nevertheless, cancer evolutionary studies have recently produced some information that can help to untie the knot. The concept of cancer as a species is not new, having its roots very far back in time with Boveri who for the first time assumed that aneuploidy, the starting point of malignant transformation, delineates cancer as a species [1]. Later, Duesberg and Rasnick [2], who supported the theory that cancer cells derive their complex phenotypes from random chromosome number mutation, a process that is analogous to speciation, suggested that a broad definition of species could be useful to describe some relevant features of cancer. Mark Vincent, in the same years, excellently reexamined the concept of “cancer as species” supported from the gradually recognized species distinctiveness of asexual organisms, enlarging the reach of the “cancer as species” concept [3]. To delve deeper into this thorny issue, we recommend the book “Debating Cancer: The Paradox in Cancer Research,” where the chapter “Do Different Cancers Represent Different Species?” excellently illustrates the evidence for and against the concept of cancer as a species [4]. Furthermore, Heng’s postulation of the genome architecture theory [5], which we will discuss later, conciliates cancer with biological evolution, definitely reserving a place for cancer as a species in itself. Following the assumption that cancer can be considered as a species, a second theme of this review is whether this species shows a somewhat “intelligent” behavior. A critical premise is that intelligence is here to be intended as a metaphor and not as a comparison with animal behaviors that are “symptoms” of intelligence. The metaphor of cancer as an “intelligent species” is an attempt to provide a different point of view on cancer adaptation through a process of simplification (by moving from complex cellular processes to macroscopic phenomena) and consilience (stimulating a “jumping together” of knowledge in different fields of science to converge on the same conclusion) [6]. In fact, metaphors (from the Greek *metapherein*, meaning “transference”) allow moving between different levels of knowledge, contributing to the development of meaning through boundless interpretive trajectories [7]. Moreover, metaphor has an instructive value, namely caused by the pleasure of the moment of understanding that follows surprise (Aristotles, *Ars Rhetorica*).

### About intelligence and species adaptation

Almost all definitions of intelligence agree on that, far from being a mere set of cognitive abilities (general intelligence), intelligence involves the ability to adapt to a changing environment (adaptive intelligence) [8]. From a biological perspective, intelligence is often referred as “adapting” to the environment, as the result of natural selection [9]. This adaptation (“narrow adaptation”) represents an oversimplification of biological intelligence. Cephalocarids, populating our seas since 250 million years are excellent adapters, but not “intelligent” in the sense in which we usually refer to intelligence. Still more cogent, the configuration change of hemoglobin shifting from R to T folds adapting to changes in oxygen partial pressure tells us that such a refined adaptive behavior cannot be considered as a sign of a molecular “intelligence” [10]. A broader (and more relevant for our aims) view of biological adaptation can be further defined as follows: (1) structural adaptation, i.e., the ability to change oneself shape to fit the environment; (2) changing metabolism to increase oneself fitness; (3) changing the environment; and (4) involve other individuals in a collective adaptation [11]. As already stressed in the “Abstract,” we concentrate on animal adaptive strategies, given the wide range of remarkable examples of the above-sketched points. Many birds, including parrots, belong to the first category, undergoing phenotypic shaping and increasing their beaks and limbs size in order to adapt to climate changing [12]. In some birds, even hybridization is an adaptive solution when landscape changes due to human interventions [13]. Naked mole-rats (*Heterocephalus glaber*) have the ability defined at point 2, changing metabolism to survive underground in low-oxygen environments [14]. Australian frogs (*Cyclorana australis*) belong to point 3, creating a new environment rich of mucus to prevent skin from drying out in the hot climate [15]. Other species, such as penguins in Antarctica, carry out the amazing collective adaptation of point 4, crowding together to share their warmth and survive glaciation. Table 1 reports the adaptive solutions to a changing environment employed by some animal species.

**Table 1:**
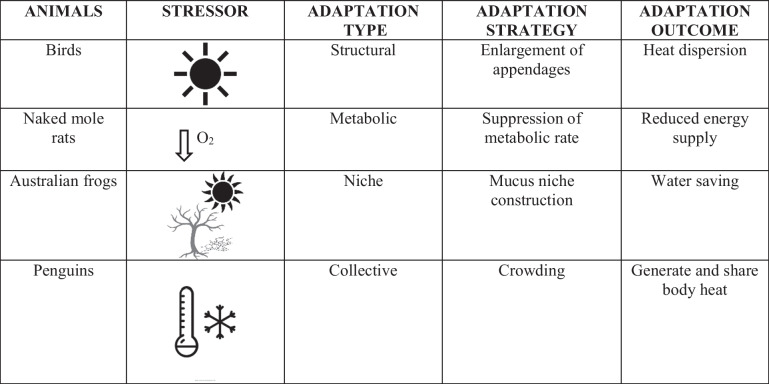
Adaptive solutions to changing environments in different animal species

In a broader sense, adaptation (and the corresponding implication of biological intelligence) also means rapidly changing oneself when moving from one environment to a different one. Animal species can react to an environmental change in three ways: moving, adapting, or dying [16]. Moving is not always easy, since colonizing a new territory means competition for spaces and resources with unfamiliar species. Many species are able to adapt to new environmental conditions through phenotypic plasticity—the ability of an organism to modify its behavioral and physical features in response to changes in the environment. Coral reefs adapted to oceans warm without any genetic change through the expulsion of the symbiotic algae that live within them [17]. Plasticity can allow genetic adaptations to conquer new habitats subsequently. Although the definition of intelligence is controversial, “adaptation to a changing environment,” “reaction to unexpected situations,” “capacity of problem solving,” and “ability to communicate” are the most frequently mentioned terms to define it [18]. Brian Ford in a fascinating article published in 2009, while reintroducing the ancient Virchow’s concept that “cell is an organism,” suggested that intelligence should be re-thinked and applied at the cell level. Cell’s behavior and interactions during inflammation or wound repair are suggestive that living cells can display an intelligent behavior since they have remarkable abilities to make decisions and take constructive actions, independently from brain regulation [19]. Cells usually know how to be compliant in a community, respecting the constraints imposed by the microenvironment. Cells know how to be autonomous while responding to stressors: hypertrophy and hyperplasia are just two examples of how normal cells adapt to damage, as a further demonstration that they are often ingenious, being able to solve problems in unexpected situations [20]. Cells communicate with each other by autocrine, paracrine, endocrine, and contact-mediated signalling pathways, which are essential to maintain and promote homeostasis. A very peculiar category of cells is represented by invasive cancer cells, which move from native to distant sites of the body thus being continuously exposed to new environmental conditions [21]. Even cancer cells, similarly to animal species, can react to an environmental change in three ways: moving, adapting, or dying. The choice is often dictated by the fitness of the single tumor cell through the interaction of its own phenotype with the local environmental conditions. As an example, hypoxic tumor regions characterized by low perfusion might support the survival of few, more fit cancer cells, able to adapt to low oxygen concentrations by activating programs controlling glycolysis, angiogenesis, invasion, immune suppression, and treatment resistance. Conversely, hypoxia will induce detachment of unfit tumor cells from the primary tumor mass [22, 23]. Whatever the stimulus that causes the detachment of tumor cells from the primitive mass, once disconnected, cancer cells enter into the bloodstream, becoming circulating tumor cells (CTCs) [24]. Blood represents the first, extremely hostile environment that a cancer cell encounters once detached, so that CTCs must rapidly carry specific adaptations to survive to potentially destructive shearing forces to evade the immune system surveillance and to resist to anticancer drugs. In order to complete the metastatic program, CTCs must rapidly adapt to this new habitat, react to unexpected situations, and communicate with the new blood neighbors, as well as with the tissue-resident neighbors living in pre-metastatic sites [25]. In other words, CTCs have to use a complex and “intelligent” adaptation strategy to survive. The aim of this review was to deepen the adaptation mechanisms of cancer cells to the blood microenvironment metaphorically referring to four adaptation strategies used by animal species: phenotypic adaptation, metabolic adaptation, niche adaptation, and collective adaptation.

### Phenotypic adaptation to stressors: birds and circulating cancer cells as “shape-shifters”

**Fig. 1:**
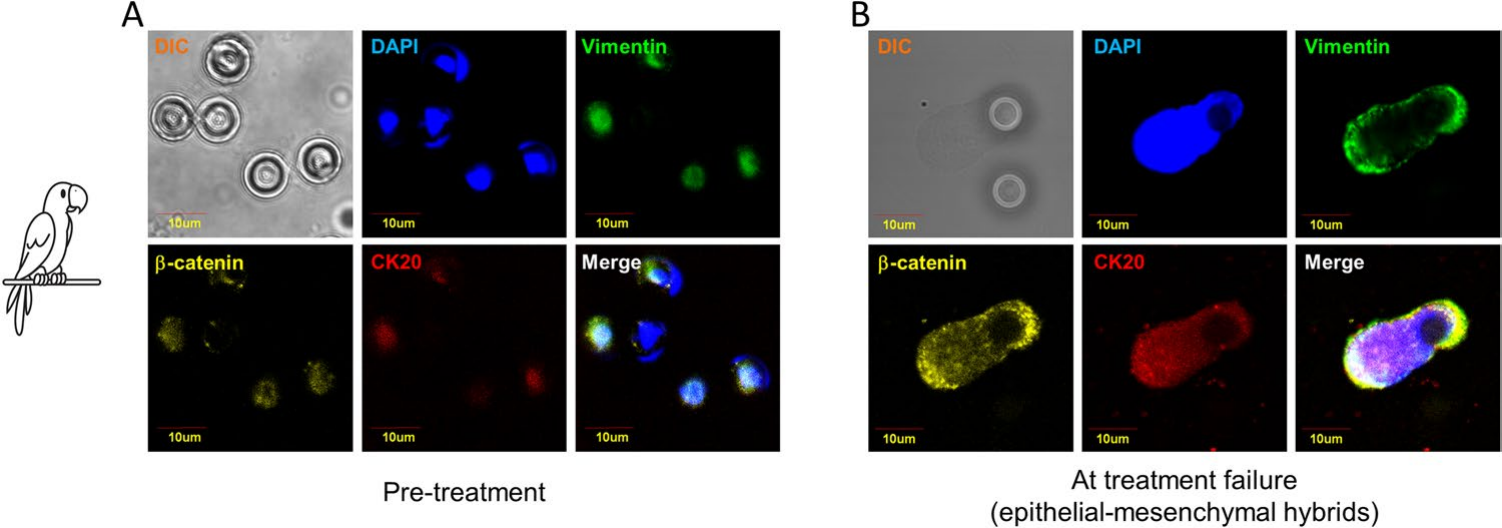
Parrot-like circulating tumor cells. EMT markers have been investigated in CTCs isolated from metastatic colorectal cancer patients in pre-treatment (panel **A**) compared to treatment failure (panel **B**). A strong upregulation of the epithelial marker CK-20 was observed at treatment failure as compared to baseline

Phenotypic plasticity is one of the mechanisms that can produce species adaptations to environmental changes. Birds are excellent examples of “shape-shifters,” having recently seen an increase in the size of all appendages such as beaks, legs, and ears due to climate change. This shape-shifting plays an important role in regulating body heat dispersion [26]. Like birds, CTCs are good example of “shape shifters,” adapting to different stressors, including anticancer drugs, through the acquisition of epithelial mesenchymal transition (EMT) features [27]. In cancer, EMT is a cellular strategy to adapt to a new environment and to react to unexpected situations. It consists in a cellular program that alters cancer cell shape, leading to an elongated form with a front-back polarity, allowing motility and survival into the circulation through the acquisition of mesenchymal traits [28]. During EMT, cytoskeletal intermediate filaments undergo a compositional change as epithelial cells, loosing keratin intermediate filaments (IFs), E-cadherin, and epithelial specific markers while acquiring mesenchymal markers such as fibronectin, N-cadherin, and vimentin [29]. Cells gain a fibroblastoid invasive phenotype, and become resistant to detachment-induced death (anoikis, as explained in detail below) [30]. Due to the typical change in IF composition, vimentin is considered a typical EMT hallmark [31]. Several lines of evidence suggest that cancer cells exploit EMT to gain resistance to several treatments, including chemo-, radio-, and immunotherapy [32]. Whether EMT features are acquired in the primary tumor or within the circulation is still controversial, although recent data suggest that—at least in some tumor types—the EMT pathway is acquired by cancer cells during the hematogenous spread [33]. Specifically, it has been suggested that mesenchymal transformation of CTCs might be mediated by transforming growth factor beta (TGF-β) released from platelets [34]. Regardless of EMT inducers, it is widely accepted that EMT represents one of the key adaptation mechanisms of CTCs to stressors, including anticancer drugs. In fact, EMT-positive CTCs have increased expression of antiapoptotic proteins and transporters belonging to ATP binding cassette family, that are responsible of drug efflux [35]. Accordingly, several studies have demonstrated that CTCs isolated from cancer patients unresponsive to standard anticancer drugs manifest EMT phenotypes [36]. A pivotal study conducted by Yu et al. suggested an association of mesenchymal CTCs with disease progression in breast cancer patients. The authors compared CTC features in pre- and post-treatment blood samples reporting an increased number of mesenchymal-like CTCs in post-treatment samples [37]. Raimondi et al. reported that CTCs isolated from patients not responding to immune checkpoint inhibitors displayed an unusual elongated spindle-like morphology compared to those isolated from responders, suggesting that elongated CTCs may represent a small population of partial EMT-transformed cancer cells [38]. Mego et al. demonstrated that CTCs undergoing EMT are associated to resistance to neoadjuvant treatments in breast cancer, suggesting a link between therapeutic stress and activation of EMT program in drug-surviving CTCs [39]. Oliveras-Ferraros et al. provided experimental evidence that EMT features in CTCs isolated from basal-like breast cancer patients recognize a new subgroup of HER2 gene-amplified breast carcinomas with primary resistance to HER2-targeted therapies, such as trastuzumab [40]. Data obtained from non-small cell lung cancer suggested that an increase of EMT markers in CTC subpopulations might be a cause of resistance to treatment with tyrosine kinase inhibitors (TKIs) [41]. It is becoming increasingly clear that carcinoma cells, rather than undergoing a full mesenchymal transformation, often attain a hybrid epithelial/mesenchymal (E/M) phenotype, also referred to as partial or incomplete EMT [42]. These cells do not completely loose epithelial features and do not completely attain mesenchymal traits, with several studies demonstrating a higher malignant potential of E/M hybrids as compared to fully mesenchymal variants [43]. The enhanced drug resistance traits of E/M hybrids as compared to fully epithelial or fully mesenchymal cells has been reported in several cancer types. Evidence has been also provided that cancer cells with EMT features need to re-upregulate epithelial markers, undergoing mesenchymal-epithelial transition (MET) immediately before colonizing distant sites. Consistently, in a population of metastatic colorectal cancer patients treated with chemotherapy and antiangiogenic drugs, we recently observed that CTCs isolated at treatment failure re-upregulated epithelial features, probably reflecting cells undergoing MET, characterized by high plasticity and strongly committed to metastatic spread (unpublished data and Fig. 1). Metaphorically speaking, the phenotypic plasticity adopted by CTCs in response to a new and hostile microenvironment is reminiscent of phenotypic alterations described in parrots under climate change.

**Fig. 2:**
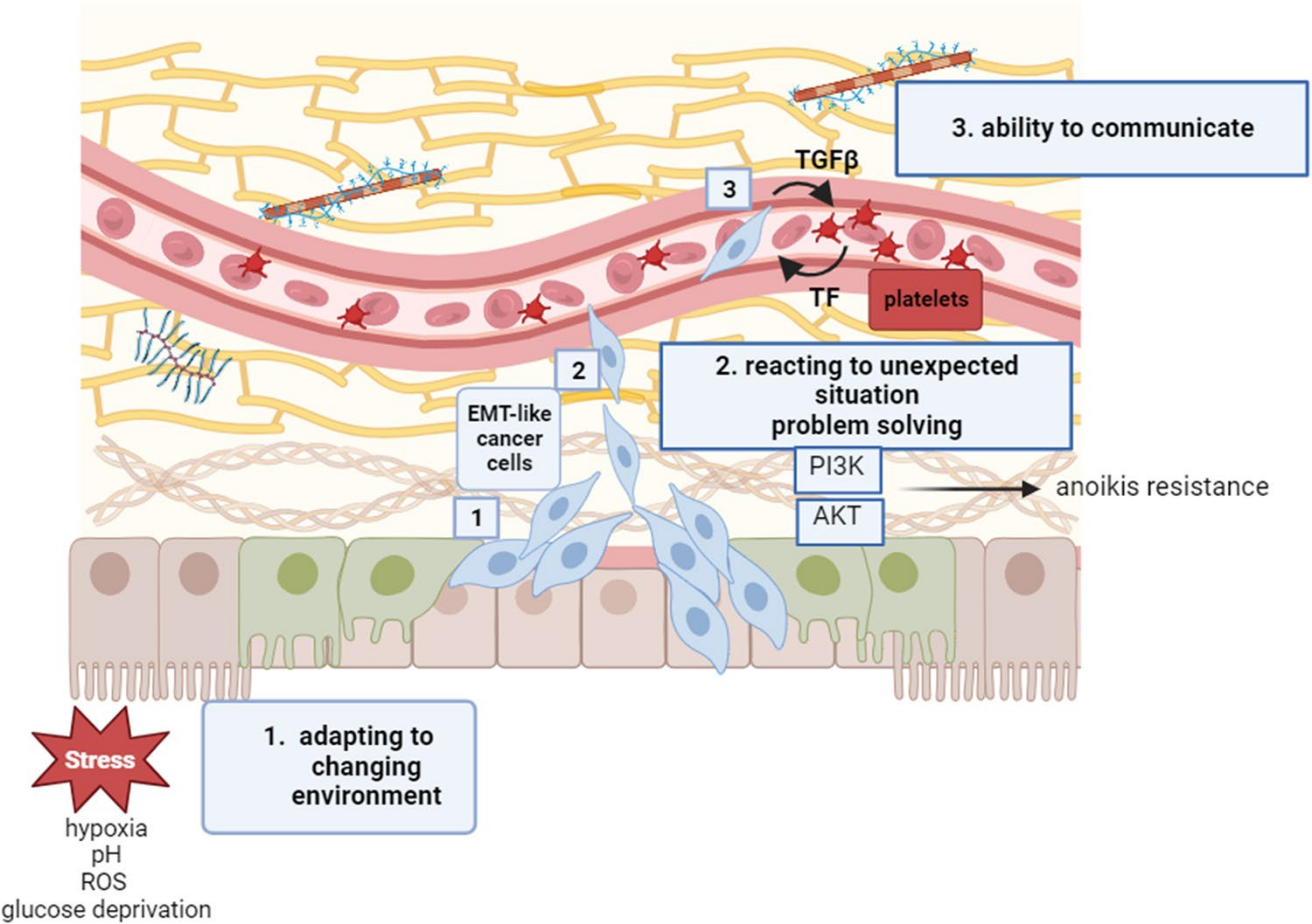
EMT as an “intelligent” behavior of cancer cells. EMT is a phenotypic adaptation of cancer cells to microenvironment stressors (1). It also represents a strategy of problem solving since the phenotypic alterations driven by EMT allow cells to elude anoikis through the activation of Akt, PI3K, or EGFR pathways (2). EMT-like CTCs are able to “communicate” with platelets through their ability to express TF, determinant for CTC survival and seeding. On the other hand, platelets are strong inducers of EMT-like CTCs through the release of TGF-β (3)

Taking into consideration the most popular signatures of intelligence (“adaptation to a changing environment,” “reaction to unexpected situations,” “capacity of problem solving,” and “ability to communicate”), EMT plasticity of circulating tumor cells seems to meet all these requirements well. In fact, the phenotypic change typical of EMT represents a cell adaptation to a changing microenvironment, such as hypoxia, acidosis, and change in glucose levels [44]. Referring to the definition of intelligence as “capacity of problem solving,” one of the main features of the EMT process is anoikis resistance [45]. Anoikis (from ancient Greek: “without a home”) is a sort of apoptotic cell death occurring upon insufficient cell-matrix interactions, representing a major problem for a cancer cell leaving tissues [46]. The dramatic phenotypic alterations driven by EMT allow cells to evade normal-tissue architectural constraints, and to escape from the primary tumor. Some CTCs through the activation of Akt, PI3K, or epidermal growth factor receptor (EGFR) pathways trigger autonomous survival mechanisms enabling EMT-shifted cells to better resist anoikis [47]. This is a clear example of how to solve a problem in a short time. Coming to the “ability to communicate,” the dynamic crosstalk between circulating tumor cells and other blood cells is increasingly recognized as a key regulator of malignant progression [48]. A large body of evidence has been provided that platelets are more than physical shields for CTCs, actively communicating with CTCs through TGF-β release to potently induce EMT phenotype [49]. CTCs “communicate” with platelets through their ability to express tissue factor (TF), determinant for CTC survival and seeding [50]. Also, CTCs have been shown to communicate with other immune cells, including neutrophils [51]. EMT induces several receptors mediating interactions between neutrophils and CTCs, including CD44, ICAM-1, and VCAM1. These results suggest that EMT-shifted CTCs are particularly efficient in communicating with platelets and neutrophils, which both support cancer cells during their journey in the bloodstream, allowing survival, resistance to shear stress, and initiation of the metastatic niche. From these reflections, one could deduce that EMT adopted by circulating cancer cells mimics an intelligent behavior (Fig. 2).

### Metabolic adaptations to stressors: naked mole-rats and circulating tumor cells as “metabolism switchers”

**Fig. 3:**
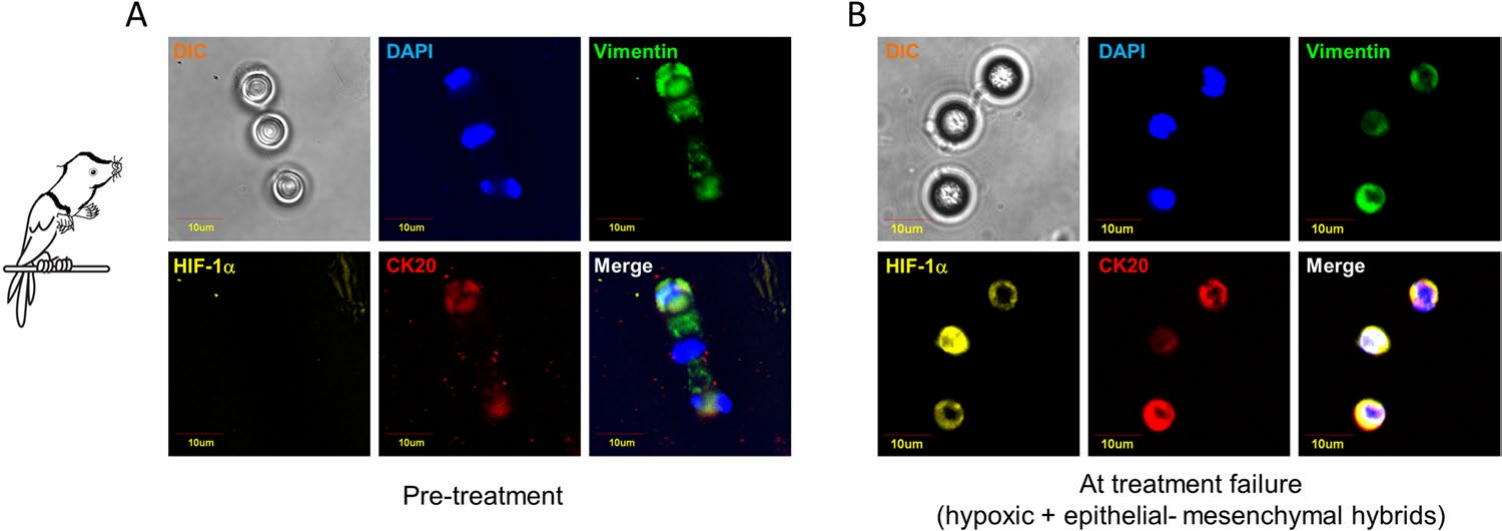
Hybrid parrots/naked mole-rats metaphorically represent circulating tumor cells. EMT and hypoxia features have been investigated in CTCs isolated from metastatic colorectal cancer patients in pre-treatment (panel **A**) compared to treatment failure (panel **B**). In panel **B**, a strong upregulation of HIF-1 compared to baseline is shown. Most CTCs maintained hybrid EMT/hypoxic characteristics

Hypoxia is one of the strongest environmental drivers of cellular metabolic adaptation [52]. Although many species are generally intolerant to hypoxia, some have evolved adaptive strategies to survive in hypoxic niches [53]. The key to tolerating hypoxia is to match metabolic demand to reduced energy supply. The naked mole-rat (*Heterocephalus glaber*) is the most hypoxia-tolerant mammal that adapted to subterranean environment remaining active despite a rapid suppression of its metabolic rate [54]. In cancer, hypoxia induces a plethora of cellular responses, including EMT induction, suppression of apoptosis, enhanced angiogenesis, malignant progression, and metabolic reprogramming, the latter allowing cell proliferation despite low-oxygen concentration [55]. Hypoxia-inducible factor 1 (HIF-1) is a key regulator of the metabolic reprogramming in hypoxic cancer cells through the regulation of genes such as the glucose transporters GLUT1 and GLUT3, hexokinase 1 and 2 (HK1 and HK2), and phosphoglycerate kinase 1 (PGK1), finally orchestrating the metabolic changes necessary to adapt to oxygen deprivation [56]. HIF is a heterodimeric complex consisting of one of the oxygen-regulated α-subunit isoforms (HIF-1α, HIF-2α, or HIF-3α) and the constitutively expressed subunit HIF-1β. While in normoxic conditions HIF-1α is regulated by hydroxylation, under hypoxia, HIF-1α hydroxylation is reduced, leading to its accumulation and nuclear translocation where the dimerization with HIF-1β occurs [57]. The HIF-1α/β dimer binds to hypoxia-response elements (HREs) of oxygen-dependent genes, including glucose transporter genes, glycolytic enzymes, and angiogenic and hematopoietic growth factors [58]. HIF-1α can upregulate pyruvate dehydrogenase kinase (PDK1), lactic dehydrogenase A (LDHA), and pyruvate kinase M2 subtype (PKM2), a key enzyme implicated in the last irreversible step of glycolysis. In addition to enhanced glycolysis, hypoxia activates the pentose phosphate pathway (PPP) [59]. HIF-1α is a common link between adaptation to hypoxia, changes in cancer metabolism, and cancer progression [60–62]. In this context, evidence has been provided that hypoxia is a major promoter of EMT through the HIF pathway, being hypoxia and EMT strictly interconnected [63]. HIF-1 induces EMT through several pathways, including TGF-β/SMAD, Wnt/beta catenin, Hedgehog, FOXM1, and through activation of EMT-specific transcription factors [64]. Notably, hypoxia can be induced by some anticancer treatments, such as antiangiogenic drugs [65]. Evidence has been provided that vascular endothelial growth factor A (VEGFA)-targeted agents such as bevacizumab promote intra-tumoral hypoxia through blocking tumor angiogenesis. In turn, hypoxia induces EMT, forces cells to abandon their native home, and reprograms the cancer stem cell niche thus favoring cancer progression [66]. Hypoxia is crucial in CTC formation, since it promotes cell invasion. Evidence has been provided that both hypoxic and normoxic cancer cells are able to intravasate [67]. Nevertheless, the survival of CTCs is strongly influenced by hypoxia, which directly stimulates the malignant properties of cancer cells through the expression of multiple genes associated with angiogenesis, metabolic regulation, cell apoptosis, and EMT [68]. Godet et al. demonstrated that cancer cells exposed to low oxygen pressure, and then to the bloodstream, acquire a “hypoxic memory or genetic signature” that is maintained even after the cells are re-oxygenated. The authors demonstrated that cancer cells exposed to physiological levels of hypoxia in the primary tumor have a 4× greater probability of becoming a viable CTC and that post-hypoxic cells have an enhanced metastasis-initiating capability [69]. A study by Kallergi et al. demonstrated that immunomagnetically isolated CTCs co-expressed VEGF and HIF-1α, suggesting that the activation of pro-angiogenic pathways in CTCs could result in evasion of apoptosis, enhancement of metastatic potential, and resistance to endocrine therapy [70]. Accordingly, undifferentiated cells expressing HIF-1α and enriched in mesenchymal phenotypes have been observed at the invasive edge of colorectal cancer [71]. Consistent with this hypothesis, our group recently observed that CTCs isolated from colorectal cancer patients who rapidly progressed in course of treatment with chemotherapy plus antiangiogenic drugs co-express HIF-1α and vimentin in response to prolonged drug exposure (unpublished data, Fig. 3). Both HIF-1α and vimentin were found consistently upregulated in CTCs at the time of treatment failure as compared to baseline. At a metaphorical level, in response to anti-angiogenic and cytotoxic treatments, CTCs implement two different adaptation strategies at the same time, i.e., hypoxia and hybrid E/M phenotypes, becoming a sort of mythological creatures half bird and half naked mole-rat.

**Fig. 4:**
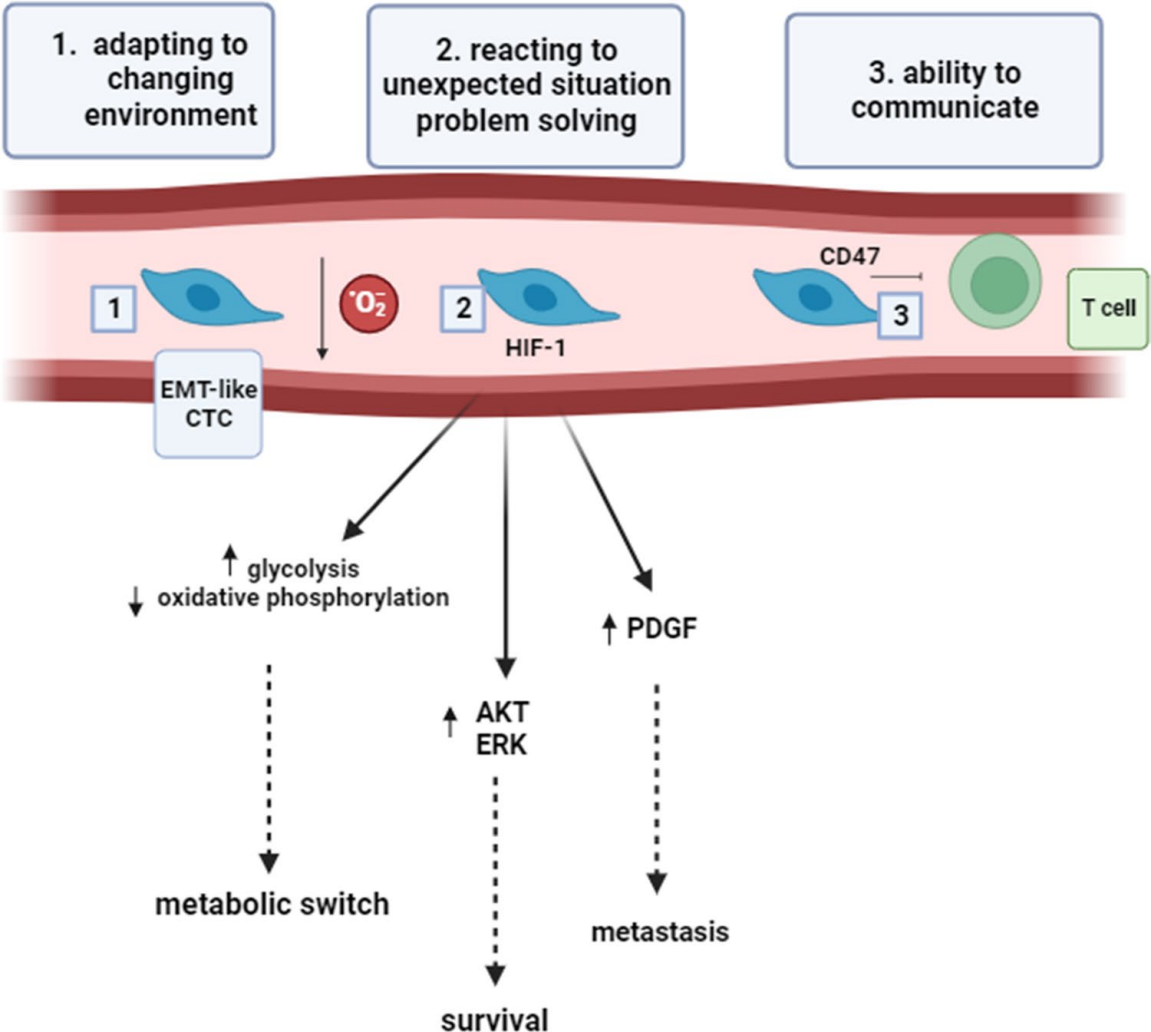
Metabolic adaptation as an “intelligent” behavior of circulating cancer cells. Hypoxia is a potent driver of EMT. Under hypoxia, CTCs maintain EMT features (1), reacting to this unexpected situation through HIF upregulation and the subsequent activation of cancer hallmarks such as metabolic switch, survival, and metastasis (2). HIF stimulates the production of CD47, which enables cancer cells to communicate with innate immune system cells in order to avoid destruction (3)

Let’s come again to the above-stressed requirements for a sensible use of intelligence metaphor: “adaptation to a changing environment,” “reaction to unexpected situations,” “capacity of problem solving,” and “ability to communicate.” Does the metabolic adaptation of CTCs meet all these requirements? The hypoxic signature of CTCs is certainly linked to a metabolic switch which represents a cell adaptation to a changing microenvironment [72]. From this point of view, the change in metabolism of the tumor cell during hypoxia represents a further example of a reaction to an unexpected situation. It is well established that hypoxic stress is a feature of most solid tumors, arising from excessive oxygen consumption by growing tumor cells and the functionally inefficient tumor-associated vasculature [73]. Hypoxia is lethal for many cancer cells that are not able to rapidly adapt to the mutated conditions. Therefore, hypoxia represents a problem that must be rapidly solved. The solution, adopted only by the most “intelligent” cells, consists in an adaptive response to hypoxia, involving HIF upregulation and the subsequent activation of cancer hallmarks such as angiogenesis, cell survival, proliferation, and metabolism switch. Coming to intelligence as “ability to communicate,” HIF has been reported to stimulate the production of CD47, a protein that enables cancer cells to avoid destruction by immune cells. Consistently, the analysis of circulating tumor cells isolated from the blood of breast cancer patients revealed that CD47 expression identified a subpopulation of cells with the capability to generate tumor xenografts in mice [74]. In the light of what has been stated in this paragraph, we postulate that the adaptation of CTCs to hypoxia is a further analogy of intelligent behavior, being part of a complex adaptation mechanism including phenotypic, metabolic, and communicative modifications (Fig. 4). Should we think of them as particularly intelligent?

### Niche adaptations to stressors: Australian frogs and circulating tumor cells as “niche organizers”

**Fig. 5:**
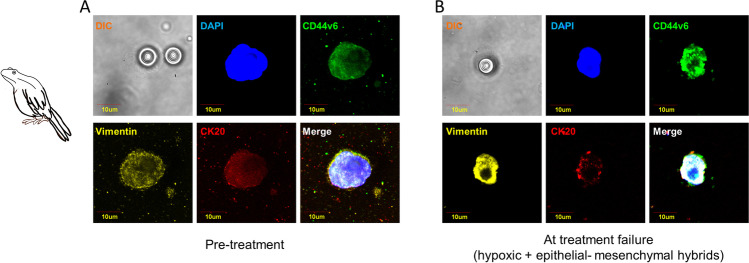
Hybrid parrots/frogs as a metaphor of circulating tumor cells. EMT and stemness markers were assessed in CTCs isolated from metastatic colorectal cancer patients in pre-treatment (panel **A**) compared to treatment failure (panel **B**). In panel **B**, a strong upregulation of CD44v6 compared to baseline is shown

The niche-construction perspective within evolutionary biology places emphasis on the changes that organisms bring about in their selective environments to adapt to stressors [75]. Australian frogs, in order to adapt to draught, enclose themselves in a transparent waterproof cocoon made from layers of shed skin until the next wet period. In this mucus niche they can stay for up to 7 months while waiting for rain. In the cancer ecosystem, a pre-metastatic niche (PMN) is defined as an environment in a secondary organ that can be conducive to the survival and outgrowth of tumor cells before their arrival at these sites [76]. Thus, in contrast to the metastatic niche that is initiated and shaped upon arrival of cancer cells, the PMN is deficient in cancer cells but conducive to their tumor growth. Both tumor-secreted factors and tumor-shed extracellular vesicles (EVs) promote the evolution of PMNs through a sequence of events, starting from vascular leakiness to the alteration of local resident fibroblasts and the further recruitment of non-resident bone marrow-derived cells (BMDCs), finally attracting circulating tumor cells [77]. The establishment of PMNs facilitates metastasis by promoting CTC survival and outgrowth. A large body of evidence demonstrate that only a small subset of circulating tumor cells are able to form metastases [78]. Although little is known about the underlying mechanisms of CTC colonization in pre-metastatic niches, the specific niche microenvironment is supposed to “educate” CTCs prior of their arrival in distant organs, contributing to the survival of tumor cells before they reach metastatic sites [79]. Tang et al. investigated the underlying mechanisms of hepatocellular carcinoma CTC colonization in pre-metastatic niches, reporting that SDF-1 in the microenvironment induces the chemotaxis of circulating CXCR4-positive CTCs to potential target organs [80]. Similarly to Australian frogs that adapt to drought until the next rain, a subpopulation of CTCs adapt to the PMN environment by acquiring enabling characteristics, until their arrival in distant metastatic sites. This subpopulation is mainly composed by cancer stem cells (CSCs) that exhibit stem-like properties and are strongly committed to efficiently colonize distant organs [81]. It has been recently suggested that CSCs and CTCs might reflect different functional states of the same subpopulation of cancer cells, being CSCs the only fraction of CTCs capable of giving rise to tumors in secondary recipients [82]. Notably, CSCs are characterized by functional plasticity for their ability to switch between mesenchymal-like and epithelial-like states; these observations led to the hypothesis that EMT is not only associated to invasive phenotype, but may also induce stemness characteristics [83]. Furthermore, CSCs have been proven to be highly resistant to standard anticancer treatments, making them a plausible cause of tumor relapse [84]. Papadaki and colleagues analyzed CTCs from breast cancer patients for CSC and EMT features, demonstrating a link between the CSC+/partial EMT and reduced progression-free survival (PFS). Interestingly, EMT-like CTCs displaying CSC features were found only in patients non-responders to chemotherapies [85]. Several lines of evidence obtained in solid tumors support the presence of CSCs with tumor-initiating capabilities within the blood [86, 87]. These cells are characterized by specific cell surface expression profiles, including CD44, CD24, CD133, CD166, and ALDH1 [88]. Gradilone et al., who investigated the expression of EMT and stemness markers in breast cancer CTCs, reported a significant association between EMT features and ALDH1 in CTCs from drug resistant patients, underlining the urgent need for optimizing CTC detection methods through the combination of EMT markers with CTC phenotype [89]. In colorectal cancer, CD44v6, the CD44 isoform mostly involved in cancer cell migration and invasion, has been identified as a functional marker of CSCs [90–93]. Nicolazzo et al. [94] provided evidence that CD44v6-positive CTCs predict treatment failure in patients with metastatic colorectal cancer undergoing first-line chemotherapy, suggesting that CD44v6-positive CTCs reflect intrinsic drug resistance in this cancer type. Consistently, we recently observed a strong upregulation of CD44v6 in CTCs isolated from colorectal cancer patients at the time of treatment failure compared to baseline (unpublished data, Fig. 5). Interestingly, all CTCs with stemness features maintained EMT traits. Again, CTCs which survived treatments implemented two different adaptation strategies in the meantime, becoming a half frog and half naked mole-rat, further supporting that EMT-related plasticity is necessary for CTCs to acquire stem-like features.

**Fig. 6:**
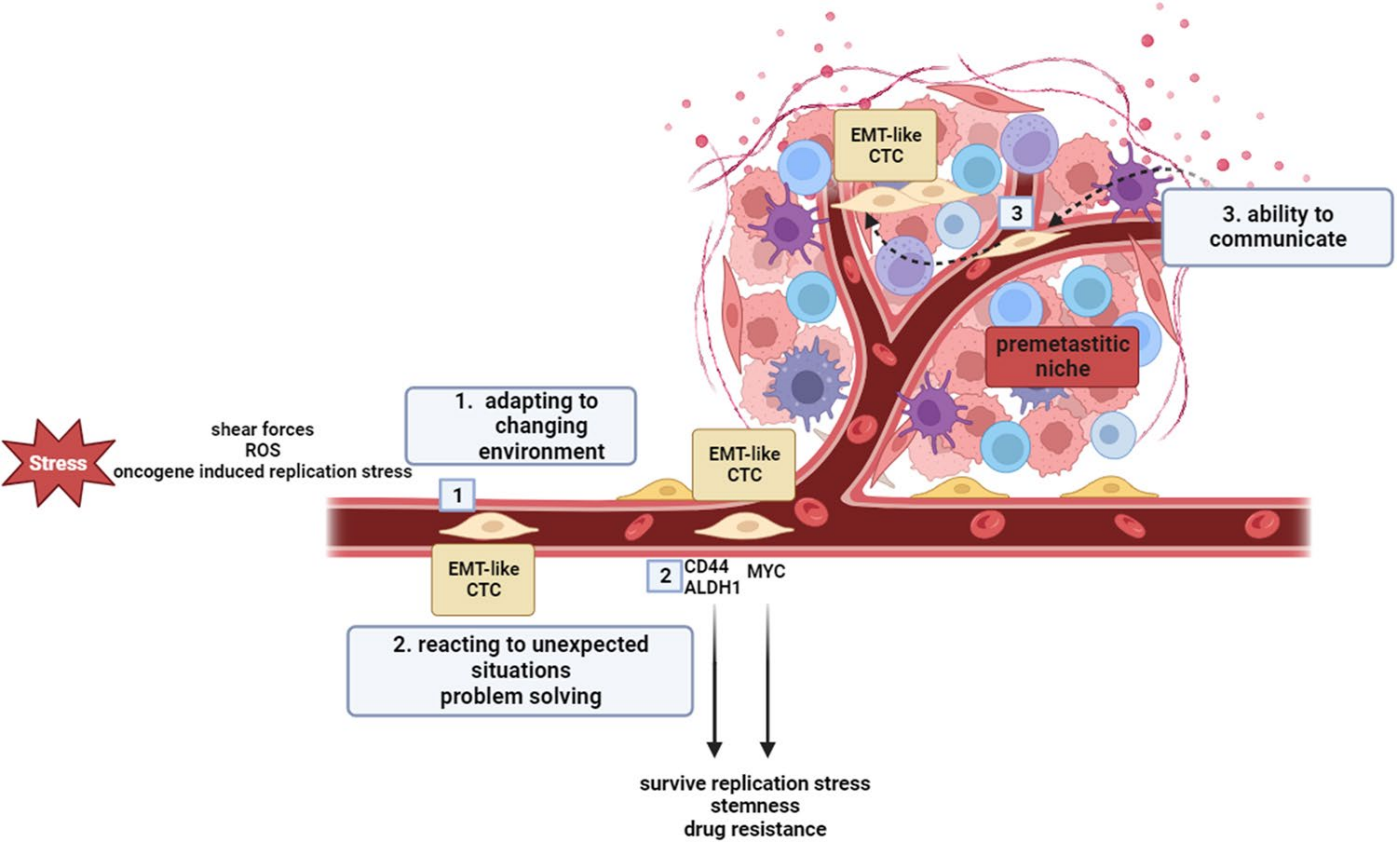
Acquisition of stemness features as an “intelligent” behavior of circulating cancer cells. Reactive oxygen species (ROS) are crucial in EMT engagement. CTCs acquire EMT plasticity when they experience oxidative stress in the bloodstream with increased metabolic demand of the mitochondria (1). CTCs react to blood stressors through MYC amplification, which allows CTCs to survive endogenous replication. Another adaptation consists in the acquisition of stemness features, which allow CTC to prepare to colonize the premetastatic niche (2). A strict crosstalk exists between circulating CSCs and immune cells, which is mediated through immune targets, as well as through EVs that enable the transfer of large biomolecular cargos among different types of cells through the release of interleukins, matrix metalloproteinases, and growth factors, finally attracting CTCs to colonize the pre-metastatic niche (3)

Let’s turn to clarify whether EMT+/CSCs can meet all the four requirements of “intelligent” behavior, i.e., “adaptation to a changing environment,” “reaction to unexpected situations,” and “capacity of problem solving.” Travelling in the bloodstream is a difficult time for CTCs. They rapidly need to adapt to various unexpected situations, specifically to all the sources of genotoxic stress (mechanical stress, oxidative stress, oncogene-induced replication stress). Evidence has been provided that CTCs experience a great amount of oxidative stress in the bloodstream with increased metabolic demand of the mitochondria, and that CTCs-specific elevated mitochondrial energy production induces a stemness gene set as an adaptive response [95]. Another difficult-to-solve problem is oncogene-induced replication stress. It has been widely reported that excess of MYC drives the cell into rapid cell cycle divisions, exacerbating multiple sources of endogenous replication stress [96]. MYC amplification is one solution to allow CTCs to survive endogenous replication stress during their journey from the primary tumor to the distant metastatic site. Consistently, MYC alterations have been described in 62% of CTCs^+^ patients. In breast cancer, MYC-expressing cells acquire CSCs and EMT features [97]. Clusters of cancer-associated fibroblasts (CAFs) together with circulating CSCs have been reported in many cancer types [98]. These complexes help CSCs to survive in the blood and probably to colonize the pre-metastatic niche. In mice, the co-injection of MCF7 cells and CAFs from a triple negative breast cancer patient led to enrichment of CSCs-like CTCs, triggering the presence of circulating CAFs/CSCs clusters [99]. Furthermore, a strict crosstalk exists between circulating CSCs and immune cells, which is mediated through immune targets, as well as through extracellular vesicles (EVs) that enable the transfer of large biomolecular cargos among different types of cells through the release of interleukins, matrix metalloproteinases, and growth factors [100]. Furthermore, it has been demonstrated that the expression of programmed cell death ligand 1 (PD-L1), which allows circulating tumor cells to elude immune attack, is often co-expressed with EMT/stemness features in CTCs, representing for these cells a possible molecular background for immune escape [38]. Altogether, the acquisition of stemness traits and PD-L1 overexpression by CTCs further reinforces the analogy of a multifaceted “intelligent” adaptation strategy (Fig. 6).

### Collective adaptations to stressors: penguins and circulating tumor cells as “social relationship builders”

**Fig. 7:**
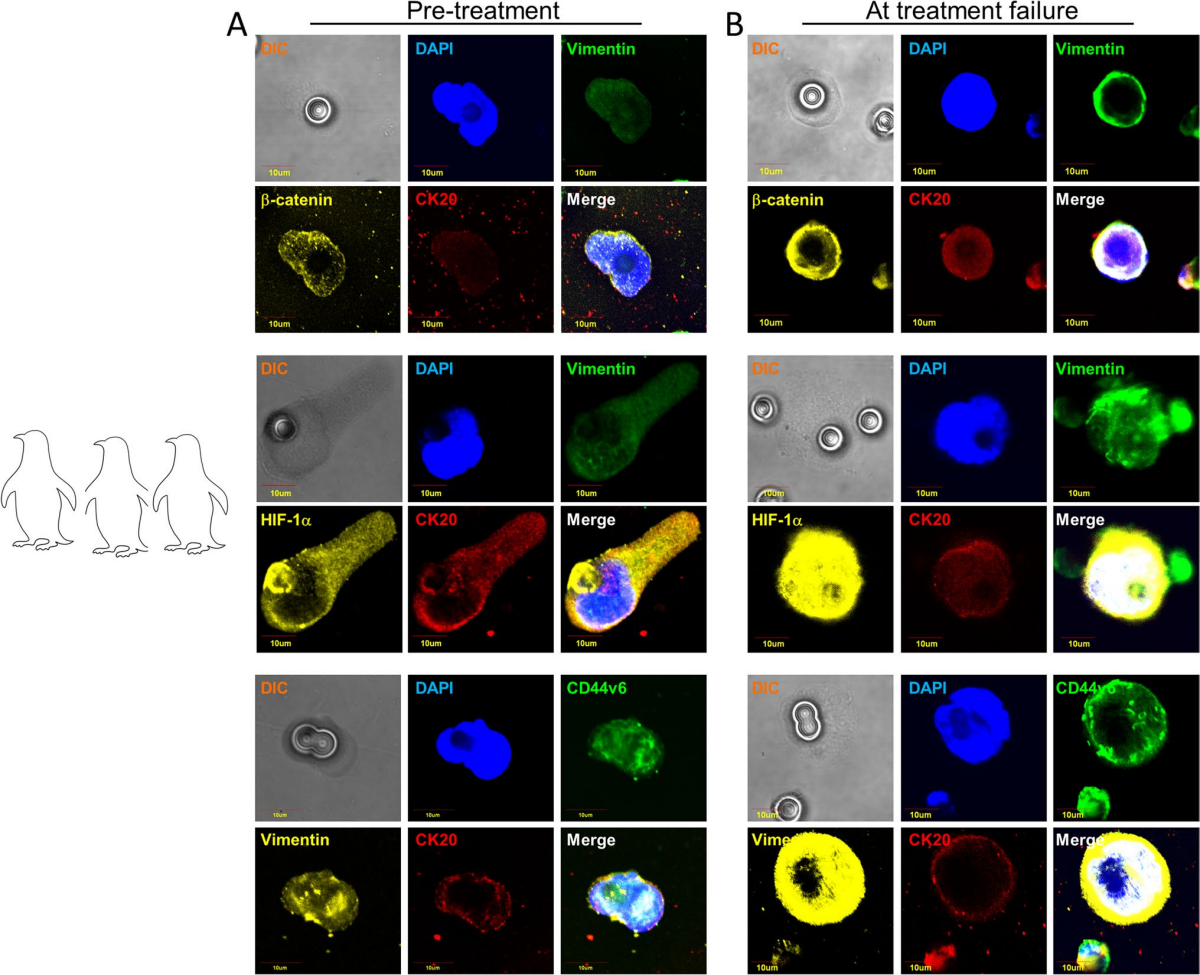
Circulating tumor clusters isolated from metastatic colorectal cancer patients remind of a penguin-like behavior. Clusters are detected at both pre-treatment (panel **A**) and at treatment failure (panel **B**)

Crowding in large groups is one of the behavioral adaptations of penguins during storms to generate and share body heat, ensuring survival in severe climate. Huddling is a sort of social thermoregulation mechanism, since the center of the huddle can reach temperatures of up to 37° Celsius [101]. Like penguins, CTCs build social relationships through cluster formation, since individual adaptation is not sufficient for a successful society [102]. Clusterization has been reported as an adaptive mechanism that enhances CTC survival in the bloodstream, and recent studies have suggested that CTC clusters, rather than being a simple aggregation of cancer cells, represent a highly specialized microenvironment [103]. A homotypic CTC cluster is defined as the aggregation of two or more cancer cells through intercellular adhesion. Cancer cells can detach from the primary site in clusters, as demonstrated by the evidence that collective migration is one of the mechanisms of invasion in cancer-host interface in human pancreatic, colorectal, lung, and breast adenocarcinomas [104]. Individual cancer cells often clump together in the blood, since single cells are more prone to apoptosis [105]. As compared to single CTCs, CTC clusters have the physical advantage to resist to anoikis and to be trapped in distant organs, thus being characterized by an increased metastasis-seeding ability [106–108]. In breast, lung, and prostate cancer, CTC clusters are reported to metastasize at 20–100 times greater efficiency and patients with higher numbers of CTC clusters have worse prognosis in terms of progression-free survival and overall survival compared to patients with single CTCs [109]. Evidence has been provided that CTC clusters better undergo margination, rotation, and adherence to endothelium than individual CTCs [110]. Several studies aimed to investigate the mechanisms of CTC clusters formation have shown that intratumor hypoxia results in cell-cell junction upregulation and intravasation of CTC clusters; specifically, in breast cancer, CTC clusters seem to derive from the hypoxic regions of the primary tumor. Another crucial trigger for cluster formation is CD44, which leads to multicellular aggregation via its target PAK2, suggesting that stemness might play a role in cluster generation through the formation of homophylic intercellular interactions [111]. Furthermore, CTC clusters contain hybrid E/M phenotypes, expressing both epithelial and mesenchymal markers. Combined stemness and EMT features might contribute to resistance to anoikis, explaining the known pro-metastatic driven properties of clustered tumor cells [112, 113]. A link between CTC clusters and anticancer drug resistance has been previously described. In ovarian cancer, the presence of CTC clusters correlates with platinum resistance [114]. Furthermore, experiments with clusters of tumor cells compartmentalized in microfluidic drops demonstrated that clusters are more resistant to doxorubicin compared to their single-cell counterparts [115]. The currently used platforms for CTC detection are not ideal for cluster isolation, while size-exclusion assays such as blood filtration represent an affordable approach for CTC cluster isolation [116, 117]. Our group recently described a new method for the simultaneous isolation of CTC clusters and single CTCs from a single blood draw through a sequential filtration, using adapted ScreenCell® filters with increased pore size. We validated the assay in a small population of patients with metastatic colorectal cancer [118]. Consistently with literature studies, CTC clusters had more prominent hybrid-EMT features compared to single CTCs; furthermore, single CTCs significantly differ from clusters in terms of HIF-1α expression, found constantly expressed in clusters, but not in single CTCs. We further investigated EMT, stemness, and hypoxic features in clusters isolated from metastatic colorectal cancer patients at pre-treatment and treatment failure (unpublished data, Fig. 7). Although clusters were detected at both timepoints, vimentin, CD44v6, and HIF-1α were found strongly upregulated at treatment failure compared to baseline.

Can clustering be viewed as an “intelligent” behavior of cancer cells? Based on current knowledge, clusters are able to outperform single CTCs in seeding metastasis with an up to 100-fold higher efficiency. Thus, the “social” behavior of CTCs can be viewed as part of CTCs “intelligent” strategy to carry out the metastatic program.

## Conclusions: why can it be productive to consider cancer as an intelligent species?

**Fig. 8:**
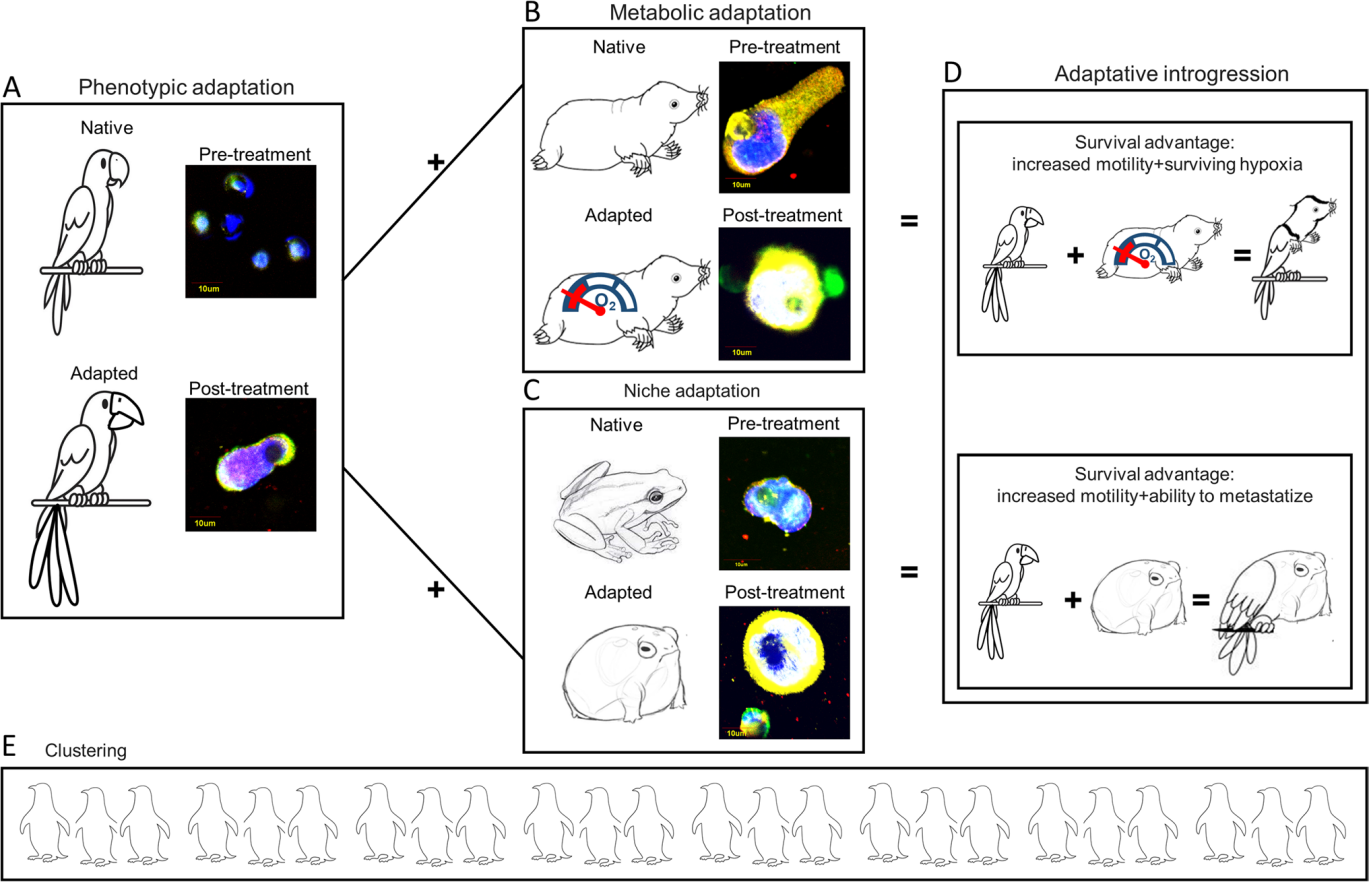
**A**–**E** Real and imaginary animal models as a metaphoric representation of structural, metabolic, niche-related, and “social” modifications of circulating cancer cells

Before going ahead and giving an answer to the most crucial question raised by this review, it is worth noting that metaphors in science are the most powerful hypothesis generators. Probably the most astonishing example of this fact is the construction of periodic table of elements by Dimitri Ivanovic Mendeleev around the half of nineteenth century. Mendeleev did not know anything about the structure of atoms (that only after more than 50 years was recognized to fit with its “octave”-based classification of elements), but he was a very good piano player. Moreover, his most strict collaborator was Aleksandr Porfirevic Borodin that, in addition to be a brilliant organic chemistry, was one of the most renowned composers of his times. Mendeleev had only knowledge of the relative weight of 32 elements and of their “valence rules,” i.e., the relative proportion of mixing into a chemical reaction so to obtain stable products. The musical background of Mendeleev pushed him to adopt the metaphorical frame of tonal music theory to order the known elements into groups substituting elements to music tones and valence rules to the interval ratios considered as “consonant” in the well-tempered scale [119]. This choice not only allowed to sketch a consistent ordination of the 32 elements known at his times but allowed to predict the position in the system and the chemical properties of the elements that were discovered in the future times. We do not think our review work can be compared to the almost unique achievement of Mendeleev; nevertheless, we wish to stress that, even if not immediately evaluable in classical quantitative terms, our concept of “cancer as an intelligent species” can be of use for scientists to interpret many puzzling phenomena of cancer development and progression. Going back to the core of our work, it is worth noting that in the bloodstream, a highly heterogeneous community circulates, since not all cells are equal [120]. This is a further demonstration of cancer biological intelligence, since an ecosystem where all individuals are identical is inexorably doomed to extinction. The fate of most CTCs in the hostile blood environment is death. Conversely, the smartest of the group quickly implement a set of different adaptation strategies, reminding a sort of mythological creatures composed by parts of different animals. It is amazing how CTCs are able to adopt more than one adaptation strategy at the same time. We have described circulating tumor cells capable of simultaneously acquiring characteristics of phenotypic and metabolic plasticity, able to increase their motility through EMT and to survive hypoxia through metabolic switch (half bird, half naked mole-rat). Others are capable of acquiring phenotypic plasticity and an extraordinary ability to adapt to specific niches to better metastasize (half bird, half Australian frog). Although hybridization is not an adaptive solution to increase fitness in most animal species (while hybridization can be a very adaptive trait in plants [121]), it provides a great opportunity to cancer cells. Donkeys and horses can breed to create mules, but hybrids are usually at risk of extinction, being sterile. Despite usually being an evolutionary dead end, sometimes hybridization introduces beneficial genes (adaptive introgression) [122]. CTCs seem more prone than animal species to something which is in some way similar to adaptive introgression. In conclusion, we provided evidence that CTCs might fulfil all the strategic steps to fall into the broader definition of biological intelligence. The required adaptations (either structural, metabolic, and related to metastatic niche formation) and “social” behavior are apparently recognized in circulating cancer cells as exemplified in the parallel behavior of several real macroscopic animal models (Fig. 8).

Coming to the presumed biological intelligence of cancer, which eventually activates a program of self-destruction causing the death of the host, one might argue that cancer behaves like a kamikaze. Is committing suicide after this enormous adaptation effort an intelligent behavior? Wouldn’t it be more successful to establish a peaceful cohabitation with its host? From a clinical perspective, establishing a long-lasting coexistence between patient and tumor may represent an intelligent therapeutic strategy [123]. The final answer to these questions will progressively emerge from the parallel evolution of tumors and anticancer treatments, predictably involving on both sides new amazing adaptation strategies.

### Re-interpreting cancer as an “intelligent” species: hints from the genome architecture theory

**Fig. 9:**
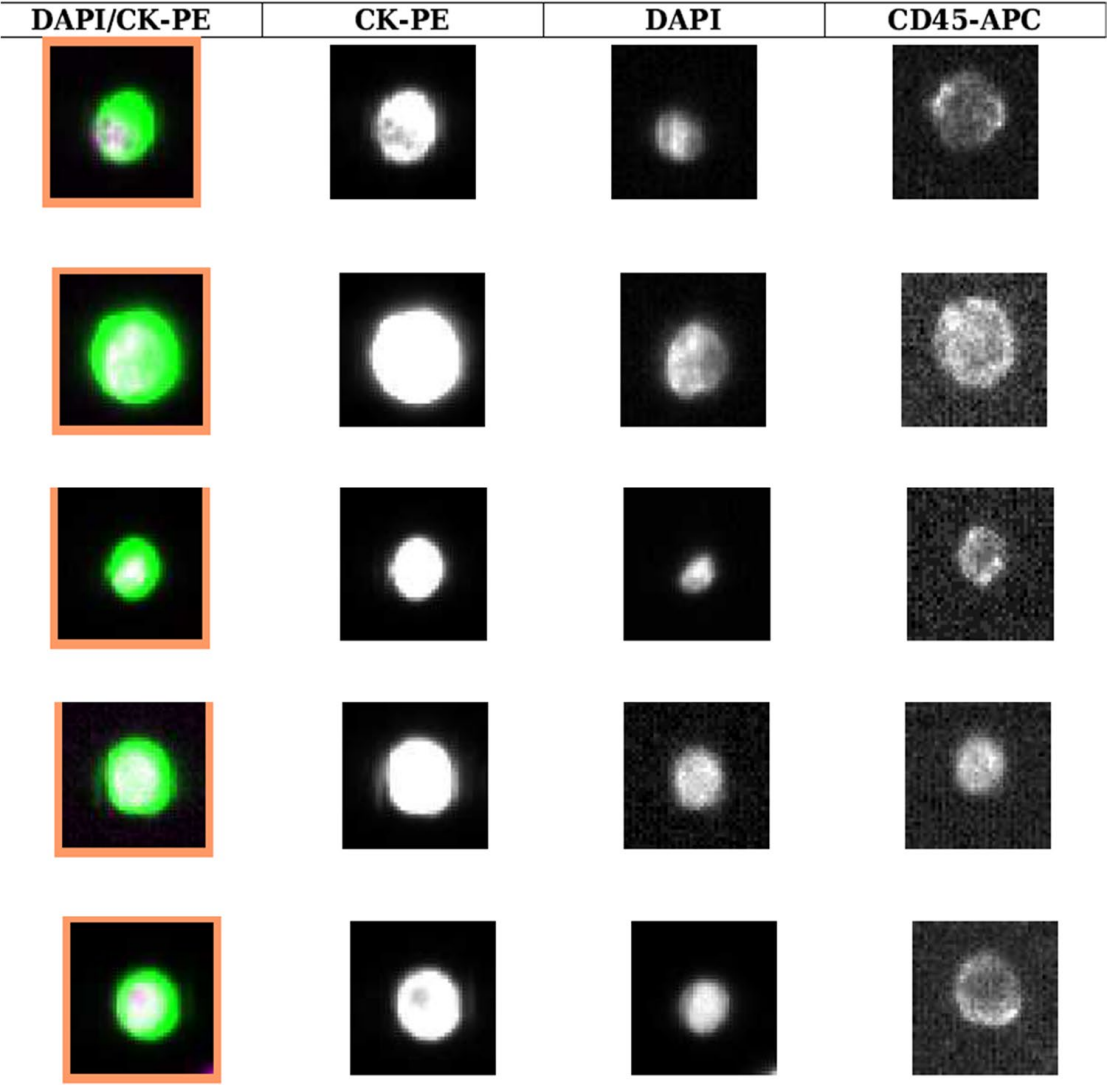
Image galleries of dual positive circulating tumor cells isolated from treatment-resistant breast cancer patients using the CellSearch platform. Immunofluorescence staining for epithelial markers (CK), leukocyte markers (CD45), and DAPI

The so-called species problem, which refers to the lack of agreement upon a clear definition of species, was recently addressed in the context of the genome architecture theory, proposed by Heng as a new tool to unify cancer with evolutionary biology strategy [5]. According to this theory, a species is a population that shares the same genome architecture. Thus, if we move from the concept of individual genes to that of the whole genome as the primary unit of heritable information, cancer represents a new species being cancer cells karyotypes totally different from those of host cells. Without neglecting the importance of individual genes in the evolutionary process, Heng postulates that the variation of single genes leads to a slow and gradual microevolution (the Darwinian theory of natural selection), while the variation of the genome leads to macroevolution which represents a sort of “explosion” able to suddenly generate new species. This “genome chaos” is the evolutionary mechanism that guides cancer progression leading to the generation of new species when the environment suddenly changes (as in course of anticancer treatments which act collectively as stressors). Thus, genome chaos can be viewed as an “intelligent” strategy to rapidly deliver macroevolutionary success by providing survivable karyotypes. This is exactly what happens in massive extinction, when a specific ecosystem suddenly is destroyed providing the opportunity for the less represented species to evolve, diversify, and become more prevalent. Although genome chaos will be lethal for some species, the most resistant will then adapt through gene mutations and epigenetic alterations with microevolution as the key feature [124]. This theory is known as the two-phased cancer evolution. According to Heng’s theory, even drug resistance is guided by genomic chaos (therefore by stress-induced macroevolution, not by microevolution) and generates new cellular species. Elegant *in vitro* studies have demonstrated that many anticancer drugs, independently from their mechanism of action, induce genome chaos resulting in the appearance of resistant clones with different structural and numerical karyotypes compared to the parental cell line. In light of this, might cancer cell species that circulate in the blood in course of treatment failure, which in this review have been ironically designed as bizarre hybrid animals, be the result of genomic chaos? To answer this question, while reading Heng’s genomic chaos theory, it occurred to us that genomic chaos in response to various genotoxic stresses, including anticancer treatments, may generate polyploid giant cancer cells (PGCCs), characterized by multiple nuclei or a single giant nucleus containing multiple complete sets of chromosomes [125]. The mechanism leading to formation of PGCCs may depend on endoreplication, mitotic slippage, cytokinesis failure, or even cell fusion [126]. Although a circulating tumor cell is classically defined as expressing epithelial markers such as EPCAM and cytokeratin (CK) and lacking the leukocyte marker CD45, the presence of CTCs with both epithelial and leukocyte markers (dual-positive cells, DP cells, CK+/CD45+) in the blood of cancer patients has recently been reported. In our experience, such hybrid CTCs are frequently detected through CellSearch platform in treatment-resistant patients (Fig. 9).

Evidence has been provided that circulating DP cells are hybrids deriving from the fusion of tumor cells and macrophages. In some tumor types, i.e., triple negative breast cancer, such hybrids present aberrant genomes and are associated with worse overall survival [127]. Indeed, several studies have demonstrated that the fusion of cancer cells with leukocytes produces hybrids with high metastatic competence by combining the epigenetic program of the leukocyte with the uncontrolled cell division of the cancer cell [128]. It is conceivable that a better characterization of DP circulating cancer cells might help to shed light on genomic chaos induced by anticancer drugs and unveil opportunities for therapeutic targeting. In conclusion, if cancer cells go through a rapid macroevolution following drug exposure, we should re-think about drug resistance as an active choice of cancer cells and not as the passive phenomenon known as treatment selection according to Darwinian natural selection theory. Thus, if we can think of drug resistance as the result of cancer evolvability through macroevolution, genome chaos might be thought as a very “intelligent” (in metaphoric sense) adaptive evolutionary strategy, allowing the formation of more aggressive cancers as a response to anticancer drugs. Such rapid, adaptive, genome-based macroevolution of cancer allows cancer cells to (1) change themselves to better fit a new environment, (2) shape the environment to better fit their needs, and (3) choose a new environment that better fits. To support our metaphor about cancer intelligence, we believe that the apparently eccentric view of cancer as an intelligent system of collaborating and computing cells (echoing the Frost [129] use of concepts borrowed by computation, game playing, and machine learning in defining cancer intelligence) deserves a thorough attention allowing to overcome some too reductionist and fatally partial views of cancer. Going back to the periodic table of elements, here we face an opposite difficulty with respect to Mendeleev: while he had to cope with a too small set of information (few chemical elements, minimal number of features) in cancer research, we have a plethora of “pieces of evidence” to be summarized into few basic concepts. A recent paper [130] demonstrates that more than 87% of human genes are somewhat related to cancer. These figures are the signature of an entropic information catastrophe: we can imagine an almost infinite number of “reliable” mechanisms of cancer if we remain linked to the usual quasi-deterministic pathway way of reasoning. This situation must be overcame by strong synthetic efforts at a more systemic scale of analysis, and the “cancer as intelligent species” hypothesis could be a promising candidate.
